# Genomic Organization and Evolution of the Trace Amine-Associated Receptor (TAAR) Repertoire in Atlantic Salmon (*Salmo salar*)

**DOI:** 10.1534/g3.114.010660

**Published:** 2014-04-22

**Authors:** Jordan A. Tessarolo, Mohammad J. Tabesh, Michael Nesbitt, William S. Davidson

**Affiliations:** Department of Molecular Biology and Biochemistry, Simon Fraser University, Burnaby, BC, V5A 1S6 Canada

**Keywords:** salmonid fishes, homing, olfaction, olfactory receptors, trace amine

## Abstract

There is strong evidence that olfaction plays a key role in the homing of salmonids to their natal spawning grounds, particularly in the freshwater phase. However, the physiological and genetic mechanisms behind this biological phenomenon are largely unknown. It has been shown that Pacific salmon respond to dissolved free amino acids from their natal streams. This indicates that amino acids comprise part of the olfcatory cues for imprinting and homing in salmonids. As trace amine-associated receptors (TAARs), a class of olfactory receptors that are close relatives of the G protein-coupled aminergic neurotransmitter receptors, recognize amino acid metabolites, we hypothesize that TAARs play an important role in salmon homing by recognizing olfactory cues. Therefore, to better understand homing in Atlantic salmon, we set out to characterize the TAAR genes in this species. We searched the first assembly of the Atlantic salmon genome for sequences resembling TAARs previously characterized in other teleosts. We identified 27 putatively functional TAAR genes and 25 putative TAAR pseudogenes, which cluster primarily on chromosome 21 (Ssa21). Phylogenetic analysis of TAAR amino acid sequences from 15 vertebrate species revealed the TAAR gene family arose after the divergence of jawed and jawless vertebrates. The TAARs group into three classes with salmon possessing class I and class III TAARs. Within each class, evolution is characterized by species-specific gene expansions, which is in contrast to what is observed in other olfactory receptor families (*e.g.*, OlfCs and oras).

Adult salmon are marveled for their ability to accurately return to their natal streams to spawn in a process known as homing. Putman *et al.* (2013) have shown recently that Pacific salmon use a geomagnetic imprinting mechanism as a means of navigating through the open ocean toward the mouth of the river, but it is hypothesized that olfaction plays a key role in salmon homing, particularly in deciphering between tributaries in the final stretch of their homeward migration (Wisby and Hasler 1951). Two main hypotheses of olfaction and homing in salmonids exist. The first, from studies involving Arctic char (*Salvelinus alpinus*) and Atlantic salmon (*Salmo salar*), suggests that the release of a population-specific pheromone by juveniles guides returning adults (Nordeng 1971, 1977). However, this cannot explain homing in chum (*Oncorhynchus keta*) and pink (*Oncorhynchus gorbuscha*) salmon because there are no juveniles present in the natal streams when the adults return. The second, and more widely accepted hypothesis, postulates odorant cues specific to different water sources are imprinted in juveniles, retained by them over the course of their ocean phase, and responded to during their upstream migration (Wisby and Hasler 1951). Additional support for the latter hypothesis comes from studies indicating that salmon are able to be imprinted, particularly during parr-smolt transformation, with a single odorant, or mixture of odorants (Shoji *et al.* 2000, 2003; Yamamoto and Ueda 2009; Yamamoto *et al.* 2010).

To understand the role of olfaction in homing in Atlantic salmon (*Salmo salar*), we set out to characterize this species’ olfactory receptor gene repertoire. We have previously shown that Atlantic salmon have seven functional and one pseudo-ora genes (Johnstone *et al.* 2008), which correspond to the vomeronasal type I genes in mammals (Saraiva and Korsching 2007), and 29 functional and 26 pseudo-OlfC genes (Johnstone *et al.* 2009), which correspond to mammalian vomeronasal type II genes (Alioto and Ngai 2006). To date, 24 putatively functional and 24 pseudo-main olfactory receptor genes have been identified (Johnstone *et al.* 2012). However, no attempt has been made to identify and characterize Atlantic salmon genes encoding trace amine-associated receptors (TAARs), the fourth class of olfactory receptors, which appears to have emerged in the common ancestor of vertebrates (Grus and Zhang 2009; Libants *et al.* 2009).

Before the discovery of TAARs, trace amines were believed to cause a symphthomimetic effect in vertebrates through competitive inhibition with neurotransmitters, such as catecholamine and serotonin (Zucchi *et al.* 2006). This point of view changed quite dramatically with the identification of a receptor class specific for these compounds. The finding by Liberles and Buck (2009) that all TAAR subtypes, except for TAAR1, are expressed in the olfactory epithelium of mice indicated that both trace amines and their receptors have roles in olfaction. Several studies suggest that the odorant cues responded to by returning Pacific salmon adults are derived from the amino acid composition of natal stream water (Shoji *et al.* 2000, 2003; Ueda 2011, 2012; Yamamoto and Ueda 2009; Yamamoto *et al.* 2010, 2013). Therefore, we hypothesize that TAARs may have an important role in salmonid imprinting and homing.

Our objectives were: (1) to identify the TAAR gene repertoire in Atlantic salmon; (2) to determine their genomic organization; (3) to examine the evolution of this gene family by a phylogenetic analysis; and (4) to predict key ligand binding site residues. We identified 27 putatively functional TAAR genes and 25 putative TAAR pseudo-genes, which cluster primarily on chromosome 21 (Ssa21). A phylogenetic analysis of TAAR amino acid sequences from 15 vertebrate species revealed that the TAAR gene family arose after the divergence of jawed and jawless vertebrates. The TAARs group into three classes, with salmon possessing class I and class III TAARs. Within each class, evolution is characterized by species-specific gene expansions, which is in contrast to what is observed in other olfactory receptor families (*e.g.*, OlfCs and oras). The amino acid positions of predicted-binding sites are well conserved across all Atlantic salmon TAARs, but the identities of these sites are only conserved within a class suggesting that TAARs within a particular class likely recognize similar molecules. The characterization of the TAAR gene family in Atlantic salmon will facilitate future studies involving olfaction and homing by enabling searches for allelic variation in different populations and changes in expression of these genes at different life stages and in salmon populations with alternative life history strategies.

## Materials and Methods

### Identification and mapping of the Atlantic salmon TAAR genes

Atlantic salmon TAAR genes and pseudogenes were identified in contig sequences from the first assembly of the Atlantic salmon genome (Davidson *et al.* 2010; http://www.ncbi.nlm.nih.gov/assembly/313068/). We used the “gene comparison” function on ASalBase (www.asalbase.org), which performs BLASTx (Altschul *et al.* 1990) of contig sequences to identify a protein of interest: in this case using *Danio rerio* TAAR 1a (ENSDARP00000012766) as the query. We classified TAARs as putatively functional if the contig had high sequence similarity to a known TAAR and contained an open reading frame of appropriate length (approximately 1 kb) in the correct reading frame and location identified through BLASTx. We termed a TAAR a putative pseudogene if there was high sequence similarity to a known TAAR but there was no obvious open reading frame or if there was a disruption in the coding sequence that was predicted to produce a truncated or abnormal product. We have temporarily named the TAAR genes/pseudogenes based on the contig in which they were identified. Only in one case did we identify two TAARs within a single contig: a pseudogene and a functional TAAR in AGKD01084249, and these were distinguished by a/b suffixes.

The TAAR genes and pseudogenes were either directly linked to the genetic map through markers within the contig, or indirectly through BLASTn of the contigs against Bacterial Artificial Chromosome clone end sequences (www.asalbase.org) or scaffolds from the Centre for Integrative Genetics. These Centre for Integrative Genetics scaffolds represent a second assembly of the Atlantic salmon genome, which have been verified and genetically mapped through SNP data (S. Lien *et al.* unpublished data). A cutoff of 98% identify over a minimum of 300 bp was applied. Matches to a Bacterial Artificial Chromosome clone end allowed the contig/TAAR to be assigned to a fingerprint scaffold (fps) on the physical map (Ng *et al.* 2005), which may or may not have been already linked with the genetic map (Danzmann *et al.* 2008) and chromosomes (Phillips *et al.* 2009) through a genetic marker (Supporting Information, Table S1). Finally, we mapped microsatellite markers derived from the contig sequences containing TAARs genes or pseudogenes using the Br5 or Br6 Atlantic salmon mapping families (Danzmann *et al.* 2008) (Table S1).

### Identification of TAARs in other vertebrates

Using recursive search techniques of NCBI and ENSEMBL databases, we were able to compile the TAAR repertoire from 14 other vertebrate species, including lamprey (*Petromyzon marinus*), elephant shark (*Callorhinchus milii*), coelacanth (*Latimeria chalumnae*), fugu (*Takifugu rubripes*), medaka (*Oryzias latipes*), stickleback (*Gasterosteus aculeatus*), tetraodon (*Tetraodon nigroviridis*), zebrafish (*Danio rerio*), frog (*Xenopus tropicalis*), alligator (*Alligator mississippiensis*), lizard (*Anolis carolinensis*), chicken (*Gallus gallus*), mouse (*Mus musculus*), and human (*Homo sapiens*). The gene count may not represent the full TAAR repertoire for these species because we only included full-length TAAR sequences in our analyses. Additional files have been provided that contain gene names, genomic positions, and accession numbers (Table S2), as well as the protein sequences in FASTA format (File S1).

### Alignment and phylogenetic analysis TAARs

**Figure 1:**
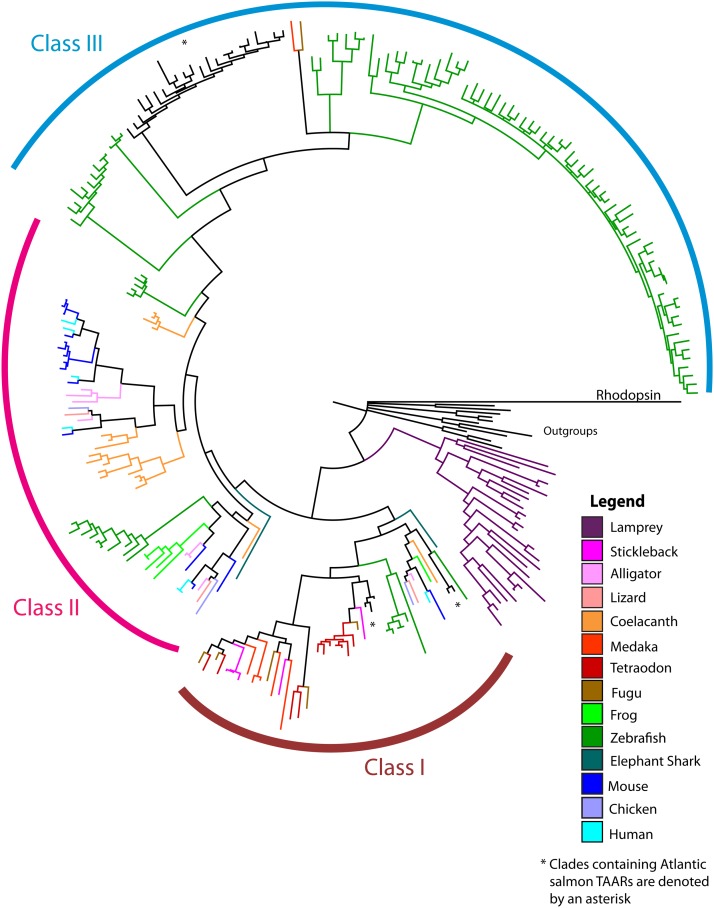
Bayesian phylogenetic analysis of 246 full-length putatively functional TAAR amino acid sequences from 15 vertebrate species including lamprey (*Petromyzon marinus*), elephant shark (*Callorhinchus milii*), coelacanth (*Latimeria chalumnae*), fugu (*Takifugu rubripes*), medaka (*Oryzias latipes*), stickleback (*Gasterosteus aculeatus*), tetraodon (*Tetraodon nigroviridis*), zebrafish (*Danio rerio*), frog (*Xenopus* tropicalis), human (*Homo sapiens*), mouse (*Mus musculus*), lizard (*Anolis carolinensis*), alligator (*Alligator mississippiensis*), chicken (*Gallus gallus*) and Atlantic salmon (*Salmo salar*) (see Table S2 for summary). Rhodopsin from human was used as an out-group. The clades containing Atlantic salmon TAARs are indicated by an asterisk. Alignment provided in File S2.

All alignments and phylogenetic analyses were carried out using Geneious 6.0.6 (Biomatters) and plugins therein. Amino acid alignments were carried out using the MUSCLE plugin (Edgar 2004). The Bayesian trees were constructed using the JTT + I + Γ model for amino acid substitution predicted by TOPALi v2. In Figure 1, we used human rhodopsin as an out-group (NM_001131055.1), and also included biogenic amine receptors including aminergic, serotonin, dopamine, and histamine H2 receptors from chicken, human and zebrafish as additional out-groups (Table S2). Following the alignment of the vertebrate TAARs, we manually removed long overhangs introduced by the out-groups and used this trimmed alignment for the phylogenetic analysis (File S2). For phylogenetic analysis of Atlantic salmon TAARs, no trimming of the amino acid alignment was done and we used zebrafish histamine receptor H2 (NM_001045338.1) as an out-group (File S3).

### Secondary structure and ligand-binding prediction

The secondary structures of the Atlantic salmon TAARs were predicted using TMHMM2.0 (Krogh *et al.* 2001). We performed this analysis on each TAAR individually as well as the consensus sequence, but we only annotated the consensus sequence (Figure S1). We used RaptorX-*binding*, a workflow of RaptorX structure prediction and DeepAlign, to predict amino acids in the ligand-binding domain (Källberg *et al.* 2012; Wang *et al.* 2013). These are annotated brown in Figure S1.

## Results

### Identification of Atlantic salmon TAAR genes and pseudo-genes

Contigs from the first assembly of the Atlantic salmon genome (Davidson *et al.* 2010; http://www.ncbi.nlm.nih.gov/assembly/313068/) were queried using the “gene comparison” function on ASalBase (www.asalbase.org) (see the *Materials and Methods* for details). We identified 27 putatively functional TAAR genes and 25 putative TAAR pseudogenes in the Atlantic salmon genome (Table S3, and Table S4). We did not name the TAARs according to the scheme proposed by Lindemann *et al.* (2005); rather, the Atlantic salmon TAARs are labeled based on the sequence contigs in which they were identified. All putatively functional TAAR genes are encoded by a single exon varying in length from 948 bp 1038 bp (Table S3).

### Genomic organization of Atlantic salmon TAAR genes and pseudogenes

The TAAR genes and pseudogenes were positioned on the Atlantic salmon physical map (Ng *et al.* 2005), the genetic map (Danzmann *et al.* 2008), or both through multiple independent techniques (see the *Materials and Methods*). We note that the multiple mapping methods utilized yielded concordant results indicating that they are robust (Table S1, Table S3, and Table S4).

The majority of mapped putatively functional TAAR genes and pseudogenes (20 of 52) are located on Ssa21 in adjacent fingerprint scaffolds: fps508 and fps943. A smaller subset (5 of 52) was found to cluster in fps798, which is located on Ssa15. A single TAAR gene or pseudogene was also found on Ssa01, Ssa02, Ssa04, Ssa13, Ssa14, with two on Ssa06. However, we were unable to map 20 of 52 TAAR genes or pseudogenes because they appear to be located in a highly complex and repetitive region of the genome making the design of polymerase chain reaction primers in the flanking regions of di- and trinucleotide repeats impossible.

### Evolution of vertebrate TAARs

To examine the evolution of these olfactory receptors, we constructed a Bayesian phylogenetic tree (Biomatters; Huelsenbeck and Ronquist 2001) (Figure 1) from an alignment of 246 full-length TAAR amino acid sequences from 15 vertebrate species, including lamprey (*Petromyzon marinus*), elephant shark (*Callorhinchus milii*), coelacanth (*Latimeria chalumnae*), fugu (*Takifugu rubripes*), medaka (*Oryzias latipes*), stickleback (*Gasterosteus aculeatus*), tetraodon (*Tetraodon nigroviridis*), zebrafish (*Danio rerio*), frog (*Xenopus tropicalis*), alligator (*Alligator mississippiensis*), lizard (*Anolis carolinensis*), chicken (*Gallus gallus*), human (*Homo sapiens*), mouse (*Mus musculus*), and Atlantic salmon (*Salmo salar*) (File S2). We used human rhodopsin as an out-group and also included biogenic amine receptors including aminergic, serotonin, dopamine, and histamine H2 receptors from chicken, human, and zebrafish as additional out-groups.

Interestingly, we found that lamprey TAARs do not cluster with the TAARs from other species. Instead, they form a species-specific clade that lies between the biogenic amine receptors and the TAARs from every other species examined.

The TAAR gene family can be separated into three main classes (class I, class II, and class III) as indentified by Hussain *et al.* (2009). The TAAR initially emerged as two classes: class I and class II. Class I TAARs form a discrete clade that contains representatives from each species examined. Class III is composed of TAARs from teleosts and evolved from the class II TAARs. Within each class there are distinct species-specific expansions.

### Phylogenetic analysis of Atlantic salmon TAARs

**Figure 2:**
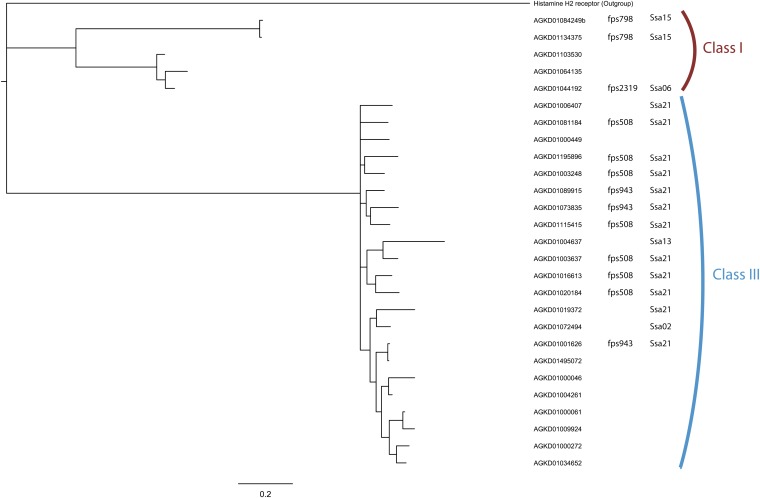
Bayesian phylogenetic analysis of 27 full-length putatively functional Atlantic salmon (*Salmo salar*) TAAR amino acid sequences using the histamine receptor H2 from *Danio rerio* as an out-group. Information regarding physical and genetic location has been added beside the contig name when available. The clades correspond to those in Figure 1. Alignment provided in File S3.

We performed Bayesian phylogenetic analysis (Biomatters; Huelsenbeck and Ronquist 2001) on the 27 putatively functional Atlantic salmon TAARs (Figure 2 and File S4). Using amino acid sequences, we found the TAARs group into three clades. The first two smaller clades correspond to class I TAARs (5 of 27), whereas the third and largest belongs to the class III teleost-specifc TAARs (22 of 27). We also added the physical and genetic mapping data to the tip labels to emphasize that the genes comprising each clade are also physically clustered together in the genome (Figure 2).

### Predicted structures of Atlantic salmon TAARs

An alignment of the *in silico* translated Atlantic salmon TAARs indicated that on average they have 66.0% pair-wise identity, with 18.9% amino acid residues being invariant among all 27 putatively functional proteins (Figure S1). All putatively functional gene products contain the TAAR fingerprint motif, NSXXNPXX[Y/H]XXX[Y/F]XWF, that was identified by (Lindemann *et al.* 2005) (Figure S1). Because the TAARs are members of the GPCR class of proteins, we used TMHMM2.0 (Krogh *et al.* 2001) to predict the secondary structures of the putatively functional salmon TAARs. Each amino acid sequence was predicted to contain seven transmembrane (TM) domains with the C-terminal domain being located in the cytosol and the N-terminal domain located extracellularly (Figure S1).

We used RaptorX-binding (Källberg *et al.* 2012; Wang *et al.* 2013) to predict which residues contribute to the ligand-binding domain. The positions of predicted ligand-binding sites were fairly uniform across the TAARs in three of the clades. The predicted binding sites were located in TM III, EL II, TM V, TM VI, and TM VII (Figure S1). The identity of these predicted ligand-binding sites are most conserved within each class; however, some sites are predicted at positions of low amino acid conservation (<30%) across all three classes.

## Discussion

### Atlantic salmon TAARs

The TAAR gene count in Atlantic salmon is comparable to the numbers reported for other teleosts except for zebrafish, which possesses by far the largest reported TAAR repertoire (Table 1). We had expected the number of TAAR genes in Atlantic salmon to be higher due to the 4R whole-genome duplication (WGD), which occurred in the common ancestor of extant salmonids (Ohno 1970; Allendorf and Thorgaard 1984). Moreover, if TAARs do indeed play a role in homing in salmon, it was anticipated that a large repertoire would be beneficial for discriminating among a wide variety of odorants. Instead, we see a large number of putative pseudogenes, which is the most likely fate of duplicated genes. It should be noted that five of the putative pseudogenes may in fact be functional, but have been temporarily classified as such due to incomplete sequence data (Table S4). The downstream signaling mechanisms initiated by these receptors are not well established, and whether a larger repertoire is required for an increase in sensitivity to specific homing cues remains unknown.

**Table 1:** Number of putatively functional TAAR genes by species included in this report compared with the previously reported number of putatively functional TAAR receptors

Species	Number of TAAR Genes in Analysis (Class I, Class II, Class III)	Database	Reported Functional TAARs	
			2007*^a^*	2009*^b^*
Chicken	3 (1, 2, 0)	NCBI	3	3
Human	7 (1, 6, 0)	NCBI	5	6
Mouse	15 (1, 14, 0)	NCBI	15	15
Frog	6 (1, 5, 0)	NCBI	6	3
Lamprey	27 (0)	Libants *et al.* 2009	21	0
Medaka	6 (5, 0, 1)	NCBI	25	25
Stickleback	5 (5, 0, 0)	Ensemble	49	48
Tetraodon	11 (11, 0, 0)	Ensemble	N/A	18
Fugu	6 (5, 0, 1)	NCBI	13	18
Zebrafish	102 (7, 15, 80)	NCBI	109	112
Coelacanth	18 (1, 17, 0)	NCBI	N/A	N/A
Lizard	3 (1, 2, 0)	NCBI	N/A	N/A
Alligator	8 (1, 7, 0)	NCBI	N/A	N/A
Elephant Shark	2 (1, 1, 0)	NCBI	N/A	2
Rat	N/A	NCBI	N/A	17
Cow	N/A	NCBI	N/A	13
Opposum	N/A	NCBI	22	19
Atlantic salmon	27 (5, 0, 22)	In House	−	−

TAARs, trace amine-associated receptors; NCBI, National Center for Biotechnology Information; N/A, not available.

a Hashiguchi and Nishida (2007).

b Hussain *et al.* (2009).

### Genomic organization of Atlantic salmon TAARs

In tetrapods, TAARs localize to a single chromosome (chromosome 10 in mouse, chromosome 6 in humans, chromosome 3 in chicken, chromosome 2 in opossum, chromosome 1 in rat) (Lindemann *et al.* 2005). The TAAR genes are mainly clustered on two chromosomes in zebrafish (chromosome 10 and chromosome 20) and also in medaka (chromosome 21 and chromosome 24) (Hussain *et al.* 2009). This finding is not surprising, given the teleost-specific WGD (Amores *et al.* 1998; Meyer and Van De Peer 2005). As salmonids have undergone an additional WGD (Allendorf and Thorgaard 1984), one might have expected salmon TAARs to cluster to four distinct genomic locations, but this pattern of organization is not seen. Clusters of Atlantic salmon TAARs are found on Ssa21 and Ssa15, but an additional seven TAARs are distributed on six different chromosomes. The Atlantic salmon genome, like those of other salmonids, has experienced extensive changes since the salmonid-specific 4R WGD as it undergoes rediploidzation from the autotetraploid state. The complex genetic organization of Atlantic salmon TAARs is likely a reflection of this ongoing genomic reorganization.

### Evolution of vertebrate TAARs

We performed phylogenetic analysis which incorporated TAARs from 15 vertebrate species, including Atlantic salmon, to elucidate the evolution of this gene family (Figure 1). Therefore, we selected species that represent the major classes within the chordate phylum: jawless fish (lamprey), carilaginous fish (elephant shark), bony fish (medaka, fugu, stickleback, zebrafish, and Atlantic salmon), lobe-finned fish (coelacanth), amphibians (frog), reptiles (lizard and alligator), birds (chicken), and mammals (mouse and human). This may not represent the complete TAAR repertoire in the species examined as we only included TAARs with full-length amino acid sequences in the analysis (see *Materials and Methods* for details). Additional effort involving functional analyses is required to annotate these genes and confirm the presence or absence of introns in this gene family. In this report, all the TAAR genes included in the phylogenetic analysis are predicted to be encoded by a single exon, except for TAAR2 from human and mouse, which have been confirmed to contain a single intron by comparisons of the genomic sequences and their transcripts (Lindemann *et al.* 2005).

The lamprey genes predicted to be TAARs did not cluster with the TAARs from the other species examined. Instead, they formed a distinct clade that rests between the biogenic amine receptors and the TAARs from every other species examined (Figure 1). This finding has two major implications. First, as suggested by Hussain *et al.* (2009), these lamprey genes are in fact not TAARs but likely some other aminergeric receptor. Second, the TAARs have likely arisen after the divergence of jawed and jawless vertebrates because every other species across vertebrate evolution was found to possess these genes to some extent. This makes the TAARs much younger from an evolutionary perspective compared to the other olfactory receptors, such as the main olfactory receptors and the vomeronasal type 1 and 2 receptors (ora and OlfC genes, respectively, in salmon).

Our results are in agreement with the finding by Hussain *et al.* (2009) that the TAAR gene family can be represented by three classes: class I, class II, and class III (Figure 1). Class I forms a discrete clade and contains representatives from each species examined including Atlantic salmon. Class I also contains TAAR1, which is the only TAAR not expressed in olfactory epithelium. Therefore, class I TAARs may be the closest representation of the ancestral TAAR gene. Class II contains TAAR genes from all species examined, except for the teleosts: medaka, tetraodon, stickleback, fugu and Atlantic salmon. Interestingly, zebrafish has maintained class II TAARs whereas these have been lost in the other teleosts examined. Finally, class III consists of TAARs only found in teleosts. They arose from class II TAARs, perhaps as a result of the teleost-specific WGD.

Within these classes, we observe species-specific expansions. This is contrary to what is seen with the other olfactory receptor classes in teleosts. For example, the genes encoding OlfCs do not form species-specific groups, but rather a species is likely to possess members from several sub-families in its repertoire with some expansions of a subfamily evident in a particular species (Johnstone *et al.* 2009). This finding suggests that the main olfactory receptors, oras and OlfCs may perform general functions required by all teleosts, and therefore representatives of all sub-families within a class have been retained to some extent. In contrast, TAARs may perform specialized functions that may be differentially selected, and therefore have evolved in a species-specific manner. This is particularly clear in zebrafish and Atlantic salmon, both of which have a large expansion of class III TAARs, suggesting these may provide a particularly important role.

### Phylogenetic analysis of Atlantic salmon TAARs

Bayesian analysis of the Atlantic salmon TAARs revealed the genes comprising each class are also physically clustered in the genome (Figure 2). Although not all of the salmon TAAR genes could be positioned on the genetic map, the trend is still clear with those that have been successfully mapped. This information will be valuable to search for additional TAAR genes when a more complete assembly of the Atlantic salmon genome becomes available (S. Lien *et al.* in preparation). Furthermore, a large expansion has occurred in the class III (teleost-specific) TAARs relative to the class I TAARs. The same observation can be made for zebrafish. Together, these results suggest additional mechanisms for gene duplication, apart from WGD, are acting on the TAARs and consequently impacting their genomic distribution and evolution.

### Predicted binding sites of TAARs

Although a few studies which have identified possible ligands for various TAARs, no crystal structures are available for any TAAR and little is known about the ligand-binding pocket (Borowsky *et al.* 2001; Bunzow *et al.* 2001; Lindemann *et al.* 2005; Zhang *et al.* 2013).

Positions of predicted-binding sites are well conserved across all Atlantic salmon TAARs, but the identity of these sites is only conserved within each class (Figure 2 and Figure S1). A small number of predicted sites are located in positions of low amino acid conservation across all Atlantic salmon TAARs, which suggests that TAARs within a class likely recognize similar molecules. Conversely, TAARs in different classes are likely to respond to different types of ligands. Although this is not surprising, identifying the ligand-binding residues will become important in future studies that will investigate whether population-specific alleles are present.

Here we report a preliminary repertoire of the TAAR genes and pseudogenes in Atlantic salmon. We identified 27 putatively functional genes (5 class I and 22 class III) and 25 putative pseudogenes that cluster primarily on Ssa21 and to a lesser extent on Ssa15. The genomic organization is reflected in the phylogenetic analysis, which gave three clades. There is conservation of predicted binding residues within each clade, but not between clades, suggesting each class of Atlantic salmon TAARs recognizes a different class of ligand. Phylogenetic analysis of TAARs across the chordate phylum reveals that these receptors arose after the divergence of jawed and jawless vertebrates. They form three classes with species-specific expansions occurring within each class. This is a different observation compared to the other olfactory receptor types. These species-specific expansions are most pronounced in zebrafish and salmon, whose expansions belong to class III TAARs. Whether this is indicative of a particular importance of these receptors for the biology of these species or simply reflects that Atlantic salmon and zebrafish are the most closely related (although distantly) among the teleosts we examined, has yet to be determined. There is increasing evidence that the odorant cues used by salmon during the final stages of homeward migration are derived from the amino acid composition of their natal stream water (Shoji *et al.* 2000, 2003; Yamamoto and Ueda 2009; Yamamoto *et al.* 2010). Therefore, receptors being capable of recognizing and distinguishing between these types of compounds would be crucial for this process to occur. The identification of the TAAR gene repertoire in Atlantic salmon will facilitate functional studies with these genes, such as their expression at different life-stages in distinct populations (Johnstone *et al.* 2011).

## Supplementary Material

Supporting Information
