# Supplementary material for: Genomic Organization and Evolution of the Trace Amine-Associated Receptor (TAAR) Repertoire in Atlantic Salmon (Salmo salar)
Source: G3 (Bethesda). 2014 Apr 22;4(6):1135–41. doi: 10.1534/g3.114.010660 (PMC4065256; doi:10.1534/g3.114.010660)
Supplement: Supporting Information [file supp_4_6_1135__index.html]

Genomic Organization and Evolution of the Trace Amine-Associated Receptor (TAAR) Repertoire in Atlantic Salmon (Salmo salar) — Supporting Information 

# Genomic Organization and Evolution of the Trace Amine-Associated Receptor (TAAR) Repertoire in Atlantic Salmon (*Salmo salar*)

## Supporting Information for Tessarolo *et al.*, 2014

**Files in this Data Supplement:**

- Supporting Information - Figure S1, Tables S1-S4, and Files S1-S3 (PDF, 279 KB)
- Figure S1 - MUSCLE alignment of full-length putatively functional Atlantic salmon TAAR amino acid sequences. Blue, pink, and red annotations below the consensus sequences denotes extracellular, intracellular, and transmembrane domains, respectively, as predicted by TMHMM. The yellow annotation above the consensus sequences marks the TAAR fingerprint motif. Brown annotations below residues within the alignment are binding site residues predicted by RaptorX-*binding*. (PDF, 8 MB)
- Table S1 - Summary of markers used for mapping including their name, source and physical/genetic location. (PDF, 132 KB)
- Table S2 - Accession numbers and genomic positions for vertebrate genes used in analyses. (PDF, 228 KB)
- Table S3 - Summary of putatively functional Atlantic salmon TAAR genes including predicted nucleotide length and physical/genetic location. (PDF, 135 KB)
- Table S4 - Summary of putative Atlantic salmon TAAR pseudogenes including physical/genetic location and predicted cause of pseudogenziation. (PDF, 135 KB)
- File S1 - All amino acid sequences used for analyses displayed in FASTA format. (.fasta, 93 KB)
- File S2 - FASTA file containing trimmed MUSCLE alignment of 246 full-length putatively functional TAAR amino acid sequences from ten vertebrate species. This alignment was used for the Bayesian tree displayed in Figure 1. (.fasta, 157 KB)
- File S3 - FASTA file containing MUSCLE alignment of 27 full-length putatively functional Atlantic salmon TAAR amino acid sequences. This alignment was used for the Bayesian tree displayed in Figure 2. (.fasta, 10 KB)
