# Supplementary material for: Genomic Organization and Evolution of the Trace Amine-Associated Receptor (TAAR) Repertoire in Atlantic Salmon (Salmo salar)
Source: G3 (Bethesda). 2014 Apr 22;4(6):1135–41. doi: 10.1534/g3.114.010660 (PMC4065256; doi:10.1534/g3.114.010660)
Supplement: Supporting Information [file supp_g3.114.010660_010660SI.pdf]

G3 · Genes | Genomes | Genetics

Genomic Organization and Evolution of the Trace Amine-Associated Receptor (TAAR) Repertoire in Atlantic Salmon (*Salmo salar*)

Jordan A. Tessarolo,[1] Mohammad J. Tabesh,[1] Michael Nesbitt,[1] and William S. Davidson[*,1]

[1]Department of Molecular Biology and Biochemistry, Simon Fraser University, Burnaby, BC, V5A 1S6 Canada

*Corresponding author: E-mail: wdavidso@sfu.ca

DOI: 10.1534/g3.114.010660

Figure S1 MUSCLE alignment of full-length putatively functional Atlantic salmon TAAR amino acid sequences. Blue, pink, and red annotations below the consensus sequences denotes extracellular, intracellular, and transmembrane domains, respectively, as predicted by TMHMM. The yellow annotation above the consensus sequences marks the TAAR fingerprint motif. Brown annotations below residues within the alignment are binding site residues predicted by RaptorX-*binding*.

Available for download as a PDF file at http://www.g3journal.org/lookup/suppl/doi:10.1534/g3.114.010660/-/DC1

Files S1-S3

Available for download at http://www.g3journal.org/lookup/suppl/doi:10.1534/g3.114.010660/-/DC1

File S1 All amino acid sequences used for analyses displayed in FASTA format.

File S2 FASTA file containing trimmed MUSCLE alignment of 246 full-length putatively functional TAAR amino acid sequences from ten vertebrate species. This alignment was used for the Bayesian tree displayed in Figure 1.

File S3 FASTA file containing MUSCLE alignment of 27 full-length putatively functional Atlantic salmon TAAR amino acid sequences. This alignment was used for the Bayesian tree displayed in Figure 2.

Table S1 Summary of markers used for mapping including their name, source and physical/genetic location. The details concerning primers and amplification conditions can be found at www.AsalBase.org.

Marker Name	Marker Source	Physical Location	Genetic Location
Ssa0907BSFU	S0152B01_SP6	fps798	Ssa15
Ssa1303BSFU	S0159E20_SP6	fps508	Ssa21
Ssa0975BSFU	S0085J07_SP6	fps943	Ssa21
Ssa0043BSFU	S0322O19_T7	fps2319	Ssa06
Ssa0014SCSFU	AGKD01073835	fps943	Ssa21
Ssa0017SCSFU	AGKD01014617	unmapped	Ssa21
Ssa0019SCSFU	AGKD01073505	unmapped	Ssa14

Table S2 Accession numbers and genomic positions for vertebrate genes used in analyses.

Organism	Gene Name	Genomic Position	Accession Number	Source
**Chicken**	Gg_TAAR1	3:56100482..56108943	XM_004935740.1	NCBI
Gallus gallus	Gg_TAAR2	3:56116829..56117857	XM_003640968.1	NCBI
	Gg_TAAR5	3:56137725..56138753	XM_001231564.2	NCBI
**Human**	Hs_TAAR6	6:132891461..132892498	NM_175067.1	NCBI
Homo sapiens	Hs_TAAR1	6:132966123..132967142	NM_138327	NCBI
	Hs_TAAR2v1	6:132938289..132945414	NM_001033080.1	NCBI
	Hs_TAAR2v2	6:132938289..132945414	NM_014626.3	NCBI
	Hs_TAAR5	6:132909731..132910877	NM_003967.2	NCBI
	Hs_TAAR8	6:132873832..132874860	NM_053278.1	NCBI
	Hs_TAAR9	6:132859427..132860475	NM_175057.3	NCBI
**Lamprey**	STIG_44993.TAAR325			Libants et al. (2009)
Petromyzon marinus	STIG_38630.TAAR335			Libants et al. (2009)
	STIG_28657.TAAR352			Libants et al. (2009)
	STIG_22166.TAAR354			Libants et al. (2009)
	STIG_17331.TAAR351			Libants et al. (2009)
	STIG_16230.TAAR353			Libants et al. (2009)
	STIG_15712.TAAR345			Libants et al. (2009)
	STIG_15153.TAAR369			Libants et al. (2009)
	STIG_15153.TAAR353			Libants et al. (2009)
	STIG_14718.TAAR353			Libants et al. (2009)
	STIG_14718.TAAR345			Libants et al. (2009)
	STIG_14409.TAAR352			Libants et al. (2009)
	STIG_12707.TAAR348			Libants et al. (2009)
	STIG_9755.TAAR355B			Libants et al. (2009)
	STIG_9755.TAAR355A			Libants et al. (2009)
	STIG_9665.TAAR351			Libants et al. (2009)
	STIG_7446.TAAR346			Libants et al. (2009)
	STIG_6636.TAAR354			Libants et al. (2009)
	STIG_5267.TAAR362			Libants et al. (2009)
	STIG_3721.TAAR365			Libants et al. (2009)
	STIG_3721.TAAR351B			Libants et al. (2009)
	STIG_3721.TAAR351A			Libants et al. (2009)
	STIG_2594.TAAR358			Libants et al. (2009)
	STIG_2594.TAAR349			Libants et al. (2009)
	STIG_2594.TAAR340			Libants et al. (2009)
	STIG_1853.TAAR349B			Libants et al. (2009)
	STIG_1853.TAAR349A			Libants et al. (2009)
**Mouse**	Mm_TAAR1	10:23920406..23921404	NM_053205.1	NCBI
Mus musculus	Mm_TAAR2	10:23938572..23941583	NM_001007266.1	NCBI

	Mm_TAAR3	10:23949558..23950589	NM_001008429.1	NCBI
	Mm_TAAR4	10:23960494..23961537	NM_001008499.1	NCBI
	Mm_TAAR5	10:23970706..23971719	NM_001009574.1	NCBI
	Mm_TAAR6	10:23984609..23985646	NM_001010828.1	NCBI
	Mm_TAAR7a	10:23992405..23993481	NM_001010829.1	NCBI
	Mm_TAAR7b	10:23999939..24001015	NM_001010827.1	NCBI
	Mm_TAAR7d	10:24027222..24028298	NM_001010838.1	NCBI
	Mm_TAAR7e	10:24037614..24038690	NM_001010835.1	NCBI
	Mm_TAAR7f	10:24049510..24050586	NM_001010839.1	NCBI
	Mm_TAAR8a	10:24076500..24077534	NM_001010830.1	NCBI
	Mm_TAAR8b	10:24091260..24092294	NM_001010837.1	NCBI
	Mm_TAAR8c	10:24100843..24101951	NM_001010840.2	NCBI
	Mm_TAAR9	10:24108488..24109534	NM_001010831.1	NCBI
**Fugu**	Tr_TAAR1-like LOC101065853	scaffold_144:4873..5868	XM_003976993.1	NCBI
Takifugu rubripes	Tr_TAAR1-like LOC101068509	scaffold_2286:2698:3676	NW_004072942.1	NCBI
	Tr_TAAR1-like LOC101075198	scaffold_229:4033..5025	XM_003977307.1	NCBI
	Tr_TAAR1-like LOC101075670	8:5604511..5605530	XM_003966556.1	NCBI
	Tr_TAAR1-like LOC101076453	scaffold_682:7142..8143	XM003976898.1	NCBI
	Tr_TAAR1-like LOC101076685	scaffold_682:15010..15984	XM_003976899.1	NCBI
**Medaka**	Ol_TAAR 7e-like LOC101169023	2:26340511..26341482	XM_004066738.1	NCBI
Oryzias latipes	Ol_TAAR 1-like LOC101174969	24:10154632..10155600	XM_004083634.1	NCBI
	Ol_TAAR 1-like LOC101174242	24:10050386..10051363	XM_004083631.1	NCBI
	Ol_TAAR1-like LOC101163058	Un:2521..3516	XM_004085890.1	NCBI
	Ol_TAAR 1-like LOC101175700	24:10183943..10184941	XM_0040083637.1	NCBI
	Ol_TAAR 1-like LOC101175452	24:10173586..10174675	NC_019882.1	NCBI
**Frog**	Xt_TAAR1	scaffold_5:56592878..56593941	XM_002935811.2	NCBI
Xenopus tropicalis	Xt_TAAR4-like LOC100497949	scaffold_5:61512858..61513896	NW_004668237.1	NCBI
	Xt_TAAR4-like LOC100488258	scaffold_5:56562660..56563662	NW_004668237.1	NCBI
	Xt_TAAR4-like LOC100487949	scaffold_5:56584949..56585950	XM_002935812.2	NCBI
	Xt_TAAR4-like LOC100488107	scaffold_2751:3356..4363	XM_002935813.1	NCBI
	Xt_TAAR4-like LOC100485003	scaffold_5:56571031..56572038	XM004914870.1	NCBI
**Zebrafish**	Dr_TAAR1b	20:46197103..46198102	NM_001082904.1	NCBI
Danio rerio	Dr_TAAR10a	20:46166706..46167717	NM_001082898.1	NCBI
	Dr_TAAR10b	20:46187583..46188593	NM_001082903.1	NCBI
	Dr_TAAR10c	20:46170540..46171551	NM_001082900.1	NCBI
	Dr_TAAR10d	20:46178797..46179975	NM_001082902	NCBI
	Dr_TAAR11	20:46192811..46193795	NM_001083077.1	NCBI
	Dr_TAAR12a	20:46239853..46240885	NM_001083109.1	NCBI
	Dr_TAAR12b	20:46224045..46225059	NM_001083104.1	NCBI
	Dr_TAAR12c	20:46220574..46221591	NM_001083095.1	NCBI
	Dr_TAAR12e	20:46264043..46265058	NM_001083094.1	NCBI

Dr_TAAR12f	20:46250999..46252013	NM_001082907.1	NCBI
Dr_TAAR12g	20:46256084..46257095	NM_001082908.1	NCBI
Dr_TAAR12h	20:46271973..46273008	NM_001082909.1	NCBI
Dr_TAAR12i	20:46211225..46212248	NM_001083085.1	NCBI
Dr_TAAR12j	20:46278935..46279958	NM_001082911.1	NCBI
Dr_TAAR13b	20:46141163..46142188	NM_001083042.1	NCBI
Dr_TAAR13c	20:46122478..46123505	NM_001083040.1	NCBI
Dr_TAAR13d	20:46133038..46134063	NM_001083041.1	NCBI
Dr_TAAR13e	20:46152019..46153044	NM_001083043.1	NCBI
Dr_TAAR14a	20:46087730..46088693	NM_001083036.1	NCBI
Dr_TAAR14b	20:46555069..46556055	NM_001082884.1	NCBI
Dr_TAAR14d	20:46542282..46543268	NM_001082912.1	NCBI
Dr_TAAR14e	20:46520053..46521039	NM_001082914.1	NCBI
Dr_TAAR14f	20:46537214..46538200	NM_001082913.1	NCBI
Dr_TAAR14h	20:46073613..46074577	NM_001083093.1	NCBI
Dr_TAAR14i	20:46069677..46070637	NM_001083037.1	NCBI
Dr_TAAR14j	20:46064040..46065004	NM_001083076.1	NCBI
Dr_TAAR14l	20:46079286..46080243	NM_001083103.1	NCBI
Dr_TAAR15	20:46117709..46118695	NM_001083039.1	NCBI
Dr_TAAR16a	10:41959548..41960522	NC_007121.5	NCBI
Dr_TAAR16f	10:41977904..41978899	NC_007121.5	NCBI
Dr_TAAR16g	10:41982677..41983672	NC_007121.5	NCBI
Dr_TAAR17a	10:41964902..41965909	XM_002663646.2	NCBI
Dr_TAAR18a	10:41889815..41890881	XM_001921119.3	NCBI
Dr_TAAR18c	10:41918143..41919165	XM_002663638.2	NCBI
Dr_TAAR18d	10:41897228..41898286	NC_007121.5	NCBI
Dr_TAAR18g	10:41894347..41895340	NC_007121.5	NCBI
Dr_TAAR18h	10:41927773..41929222	XM_002663640.3	NCBI
Dr_TAAR18i	10:41934817..41935885	NC_007121.5	NCBI
Dr_TAAR18j	10:41950041..41951072	XM_002663643.2	NCBI
Dr_TAAR19a	10:42103621..42104682	NC_007121.5	NCBI
Dr_TAAR19b	10:42140351..42141356	NC_007121.5	NCBI
Dr_TAAR19c	10:42147157..42148218	NC_007121.5	NCBI
Dr_TAAR19d	10:42134308..42135369	XM_001921481.1	NCBI
Dr_TAAR19e	10:42108791..42109853	NC_007121.5	NCBI
Dr_TAAR19h	10:42050839..42051900	NC_007121.5	NCBI
Dr_TAAR19i	10:42097939..42099000	NC_007121.5	NCBI
Dr_TAAR19j	10:42092480..42093541	NC_007121.5	NCBI
Dr_TAAR19m	10:42056442..42057541	XM_001342973.2	NCBI
Dr_TAAR19n	10:42066521..42067570	NC_007121.5	NCBI
Dr_TAAR19o	10:42043558..42044629	NC_007121.5	NCBI

Dr_TAAR19p	10:42036952..42038710	NM_001199914.1	NCBI
Dr_TAAR19q	10:42088337..42089398	NC_007121.5	NCBI
Dr_TAAR19s	10:42012869..42013930	NC_007121.5	NCBI
Dr_TAAR19t	10:42008622..42009707	NC_007121.5	NCBI
Dr_TAAR19u	10:42031263..42032312	NC_007121.5	NCBI
Dr_TAAR1-like LOC100535889	Un:6229..7240	NW_003336689.1	NCBI
Dr_TAAR20a	10:41698366..41699456	XM_001920656.2	NCBI
Dr_TAAR20a1	10:41839705..41840724	XM_002663628.2	NCBI
Dr_TAAR20b	10:41704278..41705383	XM_001920673.2	NCBI
Dr_TAAR20b1	10:41827971..41829023	NC_007121.5	NCBI
Dr_TAAR20c	Un:42..1040	BC107613.1	NCBI
Dr_TAAR20c1	10:41747212..41748268	NC_007121.5	NCBI
Dr_TAAR20d1	10:41845015..41846010	XM_002663629.2	NCBI
Dr_TAAR20f	10:41717303..41718355	NC_007121.5	NCBI
Dr_TAAR20g	10:41725305..41748271	NC_007121.5	NCBI
Dr_TAAR20h	10:41713185..41714183	XM_001341553.2	NCBI
Dr_TAAR20i	10:41731447..41732457	XM_001920721.2	NCBI
Dr_TAAR20n	10:41775937..41776989	NC_007121.5	NCBI
Dr_TAAR20o	10:41819593..41820646	NC_007121.5	NCBI
Dr_TAAR20r	10:41790993..41792046	NC_007121.5	NCBI
Dr_TAAR20u	10:41762407..41763433	XM_001920788.2	NCBI
Dr_TAAR20v	10:41808799..41809851	NC_007121.5	NCBI
Dr_TAAR20z	10:41849611..41850663	NC_007121.5	NCBI
Dr_TAAR32	10:41995697..41996758	NC_007121.5	NCBI
Dr_TAAR4-like LOC100150578	15:2040432..2041457	XM_001920809.1	NCBI
Dr_TAAR64	20:46148101..46149128	NM_001083102.1	NCBI
Dr_TAAR6-like LOC100002481	10:41834498..41835523	XM_001342220.1	NCBI
Dr_TAAR6-like LOC100002609	10:41902807..41903800	NC_007121.5	NCBI
Dr_TAAR6-like LOC100147966	10:42018541..42019952	NC_007121.5	NCBI
Dr_TAAR6-like LOC100147986	10:41790674..41791673	NC_007121.5	NCBI
Dr_TAAR9-like LOC101887086	10:41813764..41814763	NC_007121.5	NCBI
Dr_TAAR6-like LOC100150393	10:41856629..41857625	NC_007121.5	NCBI
Dr_TAAR6-like LOC100151046	10:42024091..42025099	NC_007121.5	NCBI
Dr_TAAR6-like LOC100330759	10:41910376..41911369	NC_007121.5	NCBI
Dr_TAAR6-like LOC100332495	13:305976..306950	NC_007124.5	NCBI
Dr_TAAR7a-like LOC100150419	20:46084062..46085048	NC_007131.5	NCBI
Dr_TAAR7c-like LOC100331259	10:41954907..41955899	NC_007121.5	NCBI
Dr_TAAR7d-like LOC100537518	10:42026878..42027874	NC_007121.5	NCBI
Dr_TAAR9-like LOC100151309	10:41879380..41880718	XM_002663633.2	NCBI
Dr_TAAR3-like LOC100331742	Un:10804..11790	NW_003336289.1	NCBI
Dr_TAAR2-like	20:Un:4860..5573	XM_001923755.4	NCBI

	Dr_TAAR20t	10:41767963..41769009	XM_002663626.3	NCBI
	Dr_TAAR20l	10:41771445..41772441	XM_005155417.1	NCBI
	Dr_TAAR1-like LOC100148217	10:41873631..41874632	NC_007121.5	NCBI
	TAAR 6-like LOC100148961	10:41868500..41869496	XM_001921070.3	NCBI
	Dr_TAAR20j	10:41781192..41782185	XM_002663627.2	NCBI
	TAAR 6-like LOC100149676	10:41864180..41865170	NC_007121.5	NCBI
	Dr_TAAR18b	10:41922296..41923295	XM_002663639.2	NCBI
	TAAR 9-like LOC100148476	10:41973844..41974831	NC_007121.5	NCBI
	Dr_TAAR6-like LOC100332366	13:295967..296969	NC_007124.5	NCBI
	Dr_TAAR9-like LOC100000110	13:300938..301940	NC_007124.5	NCBI
**Tetraodon**	Tn_TAAR1(6 of 15)	Un:85474851..85475831	ENSTNIT00000003225	Ensembl
Tetraodon nigroviridis	Tn_TAAR1(11 of 15)	Un:85467788..85468786	ENSTNIT00000002494	Ensembl
	Tn_TAAR1(14 of 15)	Un:35318087..35319082	ENSTNIT00000001436	Ensembl
	Tn_TAAR1(13 of 15)	Un:70258269..70259261	ENSTNIT00000007318	Ensembl
	Tn_TAAR1(15 of 15)	Un:70265697..70266692	ENSTNIT00000007319	Ensembl
	Tn_TAAR1(10 of 15)	Un: 85499769..85500764	ENSTNIT00000008083	Ensembl
	Tn_TAAR1(12 of 15)	14:1075051..1076047	ENSTNIG00000001506	Ensembl
	Tn_TAAR1(5 of 15)	14:1025315..1026317	ENSTNIG00000019179	Ensembl
	Tn_TAAR1(2 of 15)	14:1062370..1063270	ENSTNIG00000001657	Ensembl
	Tn_TAAR1(3 of 15)	14:1072819..1073800	ENSTNIG00000001360	Ensembl
	Tn_TAAR1(9 of 15)	Un:85495812..85496808	ENSTNIG00000005193	Ensembl
**Stickleback**	Ga_TAAR1(2 of 5)	groupXVIII:864898..865894	ENSGACG00000004284	Ensembl
Gasterosteus aculeatus	Ga_TAAR1(5 of 5)	groupXVIII:849379..850381	ENSGACG00000004279	Ensembl
	Ga_TAAR1(1 of 5)	groupXVIII:856337..857339	ENSGACG00000004280	Ensembl
	Ga_TAAR1(4 of 5)	groupXVIII:806425..807427	ENSGACG00000004274	Ensembl
	Ga_TAAR1(3 of 5)	groupI:27258528..2725921	ENSGACG00000015361	Ensembl
**Anole Lizard**	Ac_TAAR1	Un:4463430..4464437	XM_003223314.1	NCBI
Anolius carolinensis	Ac_TAAR2	Un:4474066..4475097	XM_003223315.1	NCBI
	Ac_TAAR5	Un:4484212..4485264	XM_003223316.1	NCBI
**American Alligator**	Am_TAAR1	Un:220473..221480	XM_006270537.1	NCBI
Alligator mississippiensis	Am_TAAR2	245497..246489	XM_006270538.1	NCBI
	Am_TAAR4-like LOC102559019	286493..287536	XM_006270529.1	NCBI
	Am_TAAR4-like LOC102558785	269855..270901	XM_006270528.1	NCBI
	Am_TAAR5-like LOC102561336	297408..298403	XM_006270539.1	NCBI
	Am_TAAR7a-like LOC102561788	335295..336326	XM_006270540.1	NCBI
	Am_TAAR9-like LOC102562260	358195..359220	XM_006270542.1	NCBI
	Am_TAAR9-like LOC102559257	305709..306737	XM_006270530.1	NCBI
**African Coelacanth**	Lc_TAAR4-like LOC102365654	Un:7900..8919	XM_006014231.1	NCBI
Latimeria chalumnae	Lc_TAAR4-like LOC102362127	Un:6731..7825	XM_006014219.1	NCBI
	Lc_TAAR9-like LOC102346275	Un:226..1260	XM_006014034.1	NCBI
	Lc_TAAR4-like LOC102346012	Un:4621..5655	XM_006014033.1	NCBI

	Lc_TAAR4-like LOC102362844	Un:16272..17312	XM_006014013.1	NCBI
	Lc_TAAR4-like LOC102358308	Un:34650..35687	XM_006013795.1	NCBI
	Lc_TAAR4-like LOC102346983	Un:43349..44395	XM_006013765.1	NCBI
	Lc_TAAR4-like LOC102362653	Un:81244..82287	XM_006013513.1	NCBI
	Lc_TAAR5-like LOC102362390	Un:53536..54543	XM_006013512.1	NCBI
	Lc_TAAR4-like LOC102361509	Un:24914..25954	XM_006013443.1	NCBI
	Lc_TAAR4-like LOC102358939	Un:98526..99563	XM_006013436.1	NCBI
	Lc_TAAR4-like LOC102367115	Un:100058..101794	XM_006013007.1	NCBI
	Lc_TAAR4-like LOC102366851	Un:54232..56373	XM_006013006.1	NCBI
	Lc_TAAR4-like LOC102366590	Un:17939..19252	XM_006013005.1	NCBI
	Lc_TAAR4-like LOC102364694	Un:107680..108717	XM_006012998.1	NCBI
	Lc_TAAR5-like LOC102348717	Un:134193..135206	XM_006011945.1	NCBI
	Lc_TAAR5-like LOC102348210	Un:47880..48881	XM_006011943.1	NCBI
	Lc_TAAR1-like LOC102360536	Un:202003..203031	XM_006010896.1	NCBI
**Elephant Shark**	Cm_contig_19181 - ORF 1 (frame 3)	Un:6852..7886	AAVX02019181.1	NCBI
Callorhinchus milii	Cm_contig_19181 - ORF 4 (frame 1)	Un:46282..47310	AAVX02019181.1	NCBI
**Atlantic Salmon**		21359..22306	AGKD01000046	ASalBase/NCBI
Salmo salar		16565..17527	AGKD01000061	ASalBase/NCBI
		35874..36836	AGKD01000272	ASalBase/NCBI
		6099..7052	AGKD01000449	ASalBase/NCBI
		2040..3002	AGKD01001626	ASalBase/NCBI
		16596..17558	AGKD01003248	ASalBase/NCBI
		13148..14107	AGKD01003637	ASalBase/NCBI
		9633..10595	AGKD01004261	ASalBase/NCBI
		12524..13471	AGKD01004637	ASalBase/NCBI
		7621..8583	AGKD01006407	ASalBase/NCBI
		8332..9279	AGKD01009924	ASalBase/NCBI
		9151..10113	AGKD01016613	ASalBase/NCBI
		3006..3971	AGKD01019372	ASalBase/NCBI
		15867..16829	AGKD01020184	ASalBase/NCBI
		10125..11087	AGKD01034652	ASalBase/NCBI
		859..1890	AGKD01044192	ASalBase/NCBI
		796..1821	AGKD01064135	ASalBase/NCBI
		1778..2743	AGKD01072494	ASalBase/NCBI
		2586..3548	AGKD01073835	ASalBase/NCBI
		3202..4164	AGKD01081184	ASalBase/NCBI
		1874..2911	AGKD01084249b	ASalBase/NCBI
		2433..3392	AGKD01089915	ASalBase/NCBI
		2628..3659	AGKD01103530	ASalBase/NCBI
		5286..6239	AGKD01115415	ASalBase/NCBI
		717..1823	AGKD01134375	ASalBase/NCBI
		9267..10224	AGKD01195896*	ASalBase/NCBI

		731..1693	AGKD01495072	ASalBase/NCBI
**OUTGROUPS**	Dr_Histamine Receptor H2	14:1618017..1619248	NM_001045338.1	NCBI
	Hs_Histamine Receptor H2	5:175084847..175136239	NM_001131055.1	NCBI
	Hs_Rhodopsin	3:129247482..129254187	NM_000539.3	NCBI
	Dr_Adrenergic Receptor Beta 3a	8:38812340..38850902	NM_001128335.1	NCBI
	Hs_Adrenergic Receptor Beta 3	8:37820513..37824184	NM_000025.2	NCBI
	Gg_Histamine Receptor H2	13:9783231..9794177	XM_004944905.1	NCBI
	Dr_Dopamine receptor D2a	15:21008238..21035946	NM_183068.1	NCBI
	Hs_Dopamine receptor D2	11:5001..70685	NM_000795.3	NCBI
	Gg_Dopamine receptor D2	24:5748287..5753356	NM_001113290.1	NCBI
	Dr_Serotonin receptor 1A b	21:19778036..19779277	NM_001145766.1	NCBI
	Hs_Serotonin receptor 1A	5:63255560..63257804	NM_000524.3	NCBI
	Gg_Serotonin receptor 1A	Z:19853067..19854401	NM_001170528.1	NCBI

*Contig with partial sequence. Complete TAAR was found in scaffold jcf2339333987_2792921-4584714_ssa21 (Lien et al., in preparation), which contains this contig

Table S3 Summary of putatively functional Atlantic salmon TAAR genes including predicted nucleotide length and physical/genetic location. Superscripts denote the various mapping methods utilized.

Contig	Length (bp)	Physical Location	Genetic Location
AGKD01000046	948	unmapped	unmapped
AGKD01000061	963	unmapped	unmapped
AGKD01000272	963	unmapped	unmapped
AGKD01000449	954	unmapped	unmapped
AGKD01001626	963	fps943[1]	Ssa21[1,2]
AGKD01003248	963	fps508[1]	Ssa21[1]
AGKD01003637	960	fps508[1]	Ssa21[1,2]
AGKD01004261	963	unmapped	unmapped
AGKD01004637	948	unmapped	Ssa13[2,3]
AGKD01006407	963	unmapped	Ssa21[2]
AGKD01009924	948	unmapped	unmapped
AGKD01016613	963	fps508[1]	Ssa21[1,2]
AGKD01019372	966	unmapped	Ssa21[2]
AGKD01020184	963	fps508[1]	Ssa21[1,2]
AGKD01034652	963	unmapped	unmapped
AGKD01044192	1026	fps2319[1]	Ssa06[1,3]
AGKD01064135	1026	unmapped	unmapped
AGKD01072494	966	unmapped	Ssa02[2]
AGKD01073835	963	fps943[1]	Ssa21[1,3]
AGKD01081184	963	fps508[1]	Ssa21[1,2]
AGKD01084249b	1038	fps798[1]	Ssa15[1,2]
AGKD01089915	960	fps943[1]	Ssa21[1,3]
AGKD01103530	1026	unmapped	unmapped
AGKD01115415	954	fps508[1]	Ssa21[1,2]
AGKD01134375	1038	fps798[1]	Ssa15[1,2]
AGKD01195896	957	fps508[1]	Ssa21[1]
AGKD01495072	963	unmapped	Unmapped

[1] BLASTn against BAC ends
[2] BLASTn against SNP mapped
[3] Microsatellite marker

Table S4 Summary of putative Atlantic salmon TAAR pseudogenes including physical/genetic location and predicted cause of pseudogenziation. Superscripts denote the various mapping methods utilized.

Name	Physical Location	Genetic Location	Predicted Cause
AGKD01001951	fps508[1]	Ssa21[1,2]	Indel / Frameshift Mutation
AGKD01036711	fps508[1]	Ssa21[1,2]	Indel / Frameshift Mutation
AGKD01087949	fps508[1]	Ssa21[1,2]	Partial match
AGKD01040598	fps798[1]	Ssa15[1,2]	Partial match
AGKD01084249a	fps798[1]	Ssa15[1,2]	Partial match
AGKD01222984	fps798[1]	Ssa15[1,2]	Nonsense mutation
AGKD01000376	unmapped	unmapped	Indel / Frameshift Mutation
AGKD01014617	unmapped	Ssa21[2,3]	Indel / Frameshift Mutation
AGKD01024914	unmapped	unmapped	Partial Match
AGKD01052335	unmapped	unmapped	Nonsense Mutation
AGKD01073505	unmapped	Ssa14[3]	Indel / Frameshift Mutation
AGKD01074920	unmapped	Ssa21[2]	Nonsense Mutation
AGKD01084072	unmapped	Ssa21[2]	Indel / Frameshift Mutation
AGKD01085575	unmapped	unmapped	Indel / Frameshift Mutation
AGKD01102951	unmapped	unmapped	more sequence data required
AGKD01137574	unmapped	Ssa06[2]	Partial Match
AGKD01143659	unmapped	unmapped	more sequence data required
AGKD01145155	unmapped	unmapped	more sequence data required
AGKD01162248	unmapped	Ssa04[2]	Partial Match
AGKD01165582	unmapped	unmapped	Frameshift Mutation / Nonsense Mutation
AGKD01284261	unmapped	Ssa01[2]	Partial Match
AGKD01437520	unmapped	Ssa21[2]	more sequence data required
AGKD01455862	unmapped	Ssa21[2]	Indel / Frameshift Mutation
AGKD01503373	unmapped	unmapped	more sequence data required
AGKD01507155	unmapped	unmapped	Partial Match

[1] BLASTn against BAC ends
[2] BLASTn against SNP mapped
[3] Microsatellite marker
