# Supplementary figures and images for: Genomic Organization and Evolution of the Trace Amine-Associated Receptor (TAAR) Repertoire in Atlantic Salmon (Salmo salar)
Source: G3 (Bethesda). 2014 Apr 22;4(6):1135–41. doi: 10.1534/g3.114.010660 (PMC4065256; doi:10.1534/g3.114.010660)

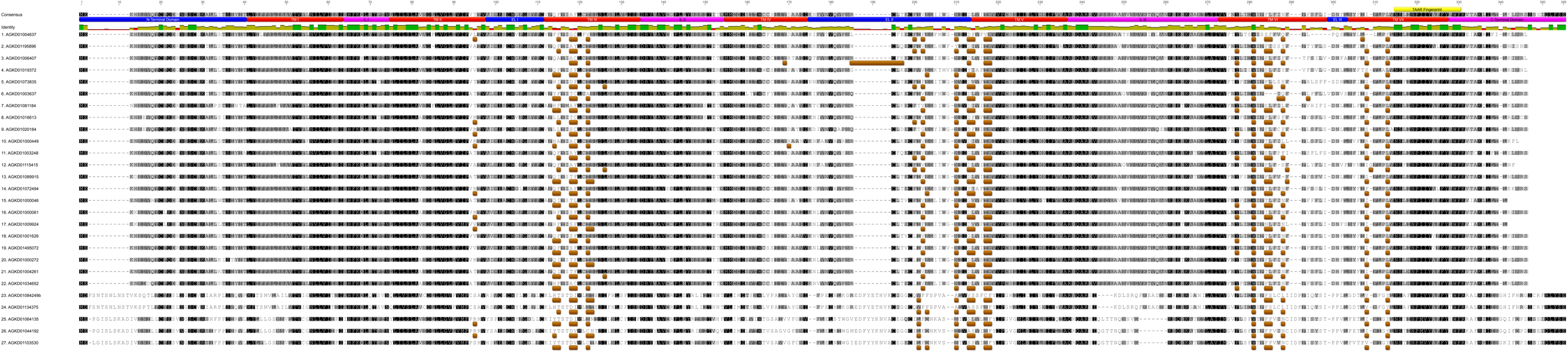

Supplement: Supporting Information [file supp_g3.114.010660_FigureS1.pdf]
