# Supplementary material for: Genomic Organization and Evolution of the Trace Amine-Associated Receptor (TAAR) Repertoire in Atlantic Salmon (Salmo salar)
Source: G3 (Bethesda). 2014 Apr 22;4(6):1135–41. doi: 10.1534/g3.114.010660 (PMC4065256; doi:10.1534/g3.114.010660)
Supplement: Supporting Information [file supp_g3.114.010660_TableS1.pdf]

Table S1 Summary of markers used for mapping including their name, source and physical/genetic location. The details concerning primers and amplification conditions can be found at www.AsalBase.org.

Marker Name	Marker Source	Physical Location	Genetic Location
Ssa0907BSFU	S0152B01_SP6	fps798	Ssa15
Ssa1303BSFU	S0159E20_SP6	fps508	Ssa21
Ssa0975BSFU	S0085J07_SP6	fps943	Ssa21
Ssa0043BSFU	S0322O19_T7	fps2319	Ssa06
Ssa0014SCSFU	AGKD01073835	fps943	Ssa21
Ssa0017SCSFU	AGKD01014617	unmapped	Ssa21
Ssa0019SCSFU	AGKD01073505	unmapped	Ssa14
