# Supplementary material for: Genomic Organization and Evolution of the Trace Amine-Associated Receptor (TAAR) Repertoire in Atlantic Salmon (Salmo salar)
Source: G3 (Bethesda). 2014 Apr 22;4(6):1135–41. doi: 10.1534/g3.114.010660 (PMC4065256; doi:10.1534/g3.114.010660)
Supplement: Supporting Information [file supp_g3.114.010660_TableS2.pdf]

Table S2 Accession numbers and genomic positions for vertebrate genes used in analyses.

Organism	Gene Name	Genomic Position	Accession Number	Source
**Chicken**	Gg_TAAR1	3:56100482..56108943	XM_004935740.1	NCBI
Gallus gallus	Gg_TAAR2	3:56116829..56117857	XM_003640968.1	NCBI
	Gg_TAAR5	3:56137725..56138753	XM_001231564.2	NCBI
**Human**	Hs_TAAR6	6:132891461..132892498	NM_175067.1	NCBI
Homo sapiens	Hs_TAAR1	6:132966123..132967142	NM_138327	NCBI
	Hs_TAAR2v1	6:132938289..132945414	NM_001033080.1	NCBI
	Hs_TAAR2v2	6:132938289..132945414	NM_014626.3	NCBI
	Hs_TAAR5	6:132909731..132910877	NM_003967.2	NCBI
	Hs_TAAR8	6:132873832..132874860	NM_053278.1	NCBI
	Hs_TAAR9	6:132859427..132860475	NM_175057.3	NCBI
**Lamprey**	STIG_44993.TAAR325			Libants et al. (2009)
Petromyzon marinus	STIG_38630.TAAR335			Libants et al. (2009)
	STIG_28657.TAAR352			Libants et al. (2009)
	STIG_22166.TAAR354			Libants et al. (2009)
	STIG_17331.TAAR351			Libants et al. (2009)
	STIG_16230.TAAR353			Libants et al. (2009)
	STIG_15712.TAAR345			Libants et al. (2009)
	STIG_15153.TAAR369			Libants et al. (2009)
	STIG_15153.TAAR353			Libants et al. (2009)
	STIG_14718.TAAR353			Libants et al. (2009)
	STIG_14718.TAAR345			Libants et al. (2009)
	STIG_14409.TAAR352			Libants et al. (2009)
	STIG_12707.TAAR348			Libants et al. (2009)
	STIG_9755.TAAR355B			Libants et al. (2009)
	STIG_9755.TAAR355A			Libants et al. (2009)
	STIG_9665.TAAR351			Libants et al. (2009)
	STIG_7446.TAAR346			Libants et al. (2009)
	STIG_6636.TAAR354			Libants et al. (2009)
	STIG_5267.TAAR362			Libants et al. (2009)
	STIG_3721.TAAR365			Libants et al. (2009)
	STIG_3721.TAAR351B			Libants et al. (2009)
	STIG_3721.TAAR351A			Libants et al. (2009)
	STIG_2594.TAAR358			Libants et al. (2009)
	STIG_2594.TAAR349			Libants et al. (2009)
	STIG_2594.TAAR340			Libants et al. (2009)
	STIG_1853.TAAR349B			Libants et al. (2009)
	STIG_1853.TAAR349A			Libants et al. (2009)
**Mouse**	Mm_TAAR1	10:23920406..23921404	NM_053205.1	NCBI
Mus musculus	Mm_TAAR2	10:23938572..23941583	NM_001007266.1	NCBI

	Mm_TAAR3	10:23949558..23950589	NM_001008429.1	NCBI
	Mm_TAAR4	10:23960494..23961537	NM_001008499.1	NCBI
	Mm_TAAR5	10:23970706..23971719	NM_001009574.1	NCBI
	Mm_TAAR6	10:23984609..23985646	NM_001010828.1	NCBI
	Mm_TAAR7a	10:23992405..23993481	NM_001010829.1	NCBI
	Mm_TAAR7b	10:23999939..24001015	NM_001010827.1	NCBI
	Mm_TAAR7d	10:24027222..24028298	NM_001010838.1	NCBI
	Mm_TAAR7e	10:24037614..24038690	NM_001010835.1	NCBI
	Mm_TAAR7f	10:24049510..24050586	NM_001010839.1	NCBI
	Mm_TAAR8a	10:24076500..24077534	NM_001010830.1	NCBI
	Mm_TAAR8b	10:24091260..24092294	NM_001010837.1	NCBI
	Mm_TAAR8c	10:24100843..24101951	NM_001010840.2	NCBI
	Mm_TAAR9	10:24108488..24109534	NM_001010831.1	NCBI
**Fugu**	Tr_TAAR1-like LOC101065853	scaffold_144:4873..5868	XM_003976993.1	NCBI
Takifugu rubripes	Tr_TAAR1-like LOC101068509	scaffold_2286:2698:3676	NW_004072942.1	NCBI
	Tr_TAAR1-like LOC101075198	scaffold_229:4033..5025	XM_003977307.1	NCBI
	Tr_TAAR1-like LOC101075670	8:5604511..5605530	XM_003966556.1	NCBI
	Tr_TAAR1-like LOC101076453	scaffold_682:7142..8143	XM003976898.1	NCBI
	Tr_TAAR1-like LOC101076685	scaffold_682:15010..15984	XM_003976899.1	NCBI
**Medaka**	Ol_TAAR 7e-like LOC101169023	2:26340511..26341482	XM_004066738.1	NCBI
Oryzias latipes	Ol_TAAR 1-like LOC101174969	24:10154632..10155600	XM_004083634.1	NCBI
	Ol_TAAR 1-like LOC101174242	24:10050386..10051363	XM_004083631.1	NCBI
	Ol_TAAR1-like LOC101163058	Un:2521..3516	XM_004085890.1	NCBI
	Ol_TAAR 1-like LOC101175700	24:10183943..10184941	XM_0040083637.1	NCBI
	Ol_TAAR 1-like LOC101175452	24:10173586..10174675	NC_019882.1	NCBI
**Frog**	Xt_TAAR1	scaffold_5:56592878..56593941	XM_002935811.2	NCBI
Xenopus tropicalis	Xt_TAAR4-like LOC100497949	scaffold_5:61512858..61513896	NW_004668237.1	NCBI
	Xt_TAAR4-like LOC100488258	scaffold_5:56562660..56563662	NW_004668237.1	NCBI
	Xt_TAAR4-like LOC100487949	scaffold_5:56584949..56585950	XM_002935812.2	NCBI
	Xt_TAAR4-like LOC100488107	scaffold_2751:3356..4363	XM_002935813.1	NCBI
	Xt_TAAR4-like LOC100485003	scaffold_5:56571031..56572038	XM004914870.1	NCBI
**Zebrafish**	Dr_TAAR1b	20:46197103..46198102	NM_001082904.1	NCBI
Danio rerio	Dr_TAAR10a	20:46166706..46167717	NM_001082898.1	NCBI
	Dr_TAAR10b	20:46187583..46188593	NM_001082903.1	NCBI
	Dr_TAAR10c	20:46170540..46171551	NM_001082900.1	NCBI
	Dr_TAAR10d	20:46178797..46179975	NM_001082902	NCBI
	Dr_TAAR11	20:46192811..46193795	NM_001083077.1	NCBI
	Dr_TAAR12a	20:46239853..46240885	NM_001083109.1	NCBI
	Dr_TAAR12b	20:46224045..46225059	NM_001083104.1	NCBI
	Dr_TAAR12c	20:46220574..46221591	NM_001083095.1	NCBI
	Dr_TAAR12e	20:46264043..46265058	NM_001083094.1	NCBI

Dr_TAAR12f	20:46250999..46252013	NM_001082907.1	NCBI
Dr_TAAR12g	20:46256084..46257095	NM_001082908.1	NCBI
Dr_TAAR12h	20:46271973..46273008	NM_001082909.1	NCBI
Dr_TAAR12i	20:46211225..46212248	NM_001083085.1	NCBI
Dr_TAAR12j	20:46278935..46279958	NM_001082911.1	NCBI
Dr_TAAR13b	20:46141163..46142188	NM_001083042.1	NCBI
Dr_TAAR13c	20:46122478..46123505	NM_001083040.1	NCBI
Dr_TAAR13d	20:46133038..46134063	NM_001083041.1	NCBI
Dr_TAAR13e	20:46152019..46153044	NM_001083043.1	NCBI
Dr_TAAR14a	20:46087730..46088693	NM_001083036.1	NCBI
Dr_TAAR14b	20:46555069..46556055	NM_001082884.1	NCBI
Dr_TAAR14d	20:46542282..46543268	NM_001082912.1	NCBI
Dr_TAAR14e	20:46520053..46521039	NM_001082914.1	NCBI
Dr_TAAR14f	20:46537214..46538200	NM_001082913.1	NCBI
Dr_TAAR14h	20:46073613..46074577	NM_001083093.1	NCBI
Dr_TAAR14i	20:46069677..46070637	NM_001083037.1	NCBI
Dr_TAAR14j	20:46064040..46065004	NM_001083076.1	NCBI
Dr_TAAR14l	20:46079286..46080243	NM_001083103.1	NCBI
Dr_TAAR15	20:46117709..46118695	NM_001083039.1	NCBI
Dr_TAAR16a	10:41959548..41960522	NC_007121.5	NCBI
Dr_TAAR16f	10:41977904..41978899	NC_007121.5	NCBI
Dr_TAAR16g	10:41982677..41983672	NC_007121.5	NCBI
Dr_TAAR17a	10:41964902..41965909	XM_002663646.2	NCBI
Dr_TAAR18a	10:41889815..41890881	XM_001921119.3	NCBI
Dr_TAAR18c	10:41918143..41919165	XM_002663638.2	NCBI
Dr_TAAR18d	10:41897228..41898286	NC_007121.5	NCBI
Dr_TAAR18g	10:41894347..41895340	NC_007121.5	NCBI
Dr_TAAR18h	10:41927773..41929222	XM_002663640.3	NCBI
Dr_TAAR18i	10:41934817..41935885	NC_007121.5	NCBI
Dr_TAAR18j	10:41950041..41951072	XM_002663643.2	NCBI
Dr_TAAR19a	10:42103621..42104682	NC_007121.5	NCBI
Dr_TAAR19b	10:42140351..42141356	NC_007121.5	NCBI
Dr_TAAR19c	10:42147157..42148218	NC_007121.5	NCBI
Dr_TAAR19d	10:42134308..42135369	XM_001921481.1	NCBI
Dr_TAAR19e	10:42108791..42109853	NC_007121.5	NCBI
Dr_TAAR19h	10:42050839..42051900	NC_007121.5	NCBI
Dr_TAAR19i	10:42097939..42099000	NC_007121.5	NCBI
Dr_TAAR19j	10:42092480..42093541	NC_007121.5	NCBI
Dr_TAAR19m	10:42056442..42057541	XM_001342973.2	NCBI
Dr_TAAR19n	10:42066521..42067570	NC_007121.5	NCBI
Dr_TAAR19o	10:42043558..42044629	NC_007121.5	NCBI

Dr_TAAR19p	10:42036952..42038710	NM_001199914.1	NCBI
Dr_TAAR19q	10:42088337..42089398	NC_007121.5	NCBI
Dr_TAAR19s	10:42012869..42013930	NC_007121.5	NCBI
Dr_TAAR19t	10:42008622..42009707	NC_007121.5	NCBI
Dr_TAAR19u	10:42031263..42032312	NC_007121.5	NCBI
Dr_TAAR1-like LOC100535889	Un:6229..7240	NW_003336689.1	NCBI
Dr_TAAR20a	10:41698366..41699456	XM_001920656.2	NCBI
Dr_TAAR20a1	10:41839705..41840724	XM_002663628.2	NCBI
Dr_TAAR20b	10:41704278..41705383	XM_001920673.2	NCBI
Dr_TAAR20b1	10:41827971..41829023	NC_007121.5	NCBI
Dr_TAAR20c	Un:42..1040	BC107613.1	NCBI
Dr_TAAR20c1	10:41747212..41748268	NC_007121.5	NCBI
Dr_TAAR20d1	10:41845015..41846010	XM_002663629.2	NCBI
Dr_TAAR20f	10:41717303..41718355	NC_007121.5	NCBI
Dr_TAAR20g	10:41725305..41748271	NC_007121.5	NCBI
Dr_TAAR20h	10:41713185..41714183	XM_001341553.2	NCBI
Dr_TAAR20i	10:41731447..41732457	XM_001920721.2	NCBI
Dr_TAAR20n	10:41775937..41776989	NC_007121.5	NCBI
Dr_TAAR20o	10:41819593..41820646	NC_007121.5	NCBI
Dr_TAAR20r	10:41790993..41792046	NC_007121.5	NCBI
Dr_TAAR20u	10:41762407..41763433	XM_001920788.2	NCBI
Dr_TAAR20v	10:41808799..41809851	NC_007121.5	NCBI
Dr_TAAR20z	10:41849611..41850663	NC_007121.5	NCBI
Dr_TAAR32	10:41995697..41996758	NC_007121.5	NCBI
Dr_TAAR4-like LOC100150578	15:2040432..2041457	XM_001920809.1	NCBI
Dr_TAAR64	20:46148101..46149128	NM_001083102.1	NCBI
Dr_TAAR6-like LOC100002481	10:41834498..41835523	XM_001342220.1	NCBI
Dr_TAAR6-like LOC100002609	10:41902807..41903800	NC_007121.5	NCBI
Dr_TAAR6-like LOC100147966	10:42018541..42019952	NC_007121.5	NCBI
Dr_TAAR6-like LOC100147986	10:41790674..41791673	NC_007121.5	NCBI
Dr_TAAR9-like LOC101887086	10:41813764..41814763	NC_007121.5	NCBI
Dr_TAAR6-like LOC100150393	10:41856629..41857625	NC_007121.5	NCBI
Dr_TAAR6-like LOC100151046	10:42024091..42025099	NC_007121.5	NCBI
Dr_TAAR6-like LOC100330759	10:41910376..41911369	NC_007121.5	NCBI
Dr_TAAR6-like LOC100332495	13:305976..306950	NC_007124.5	NCBI
Dr_TAAR7a-like LOC100150419	20:46084062..46085048	NC_007131.5	NCBI
Dr_TAAR7c-like LOC100331259	10:41954907..41955899	NC_007121.5	NCBI
Dr_TAAR7d-like LOC100537518	10:42026878..42027874	NC_007121.5	NCBI
Dr_TAAR9-like LOC100151309	10:41879380..41880718	XM_002663633.2	NCBI
Dr_TAAR3-like LOC100331742	Un:10804..11790	NW_003336289.1	NCBI
Dr_TAAR2-like	20:Un:4860..5573	XM_001923755.4	NCBI

	Dr_TAAR20t	10:41767963..41769009	XM_002663626.3	NCBI
	Dr_TAAR20l	10:41771445..41772441	XM_005155417.1	NCBI
	Dr_TAAR1-like LOC100148217	10:41873631..41874632	NC_007121.5	NCBI
	TAAR 6-like LOC100148961	10:41868500..41869496	XM_001921070.3	NCBI
	Dr_TAAR20j	10:41781192..41782185	XM_002663627.2	NCBI
	TAAR 6-like LOC100149676	10:41864180..41865170	NC_007121.5	NCBI
	Dr_TAAR18b	10:41922296..41923295	XM_002663639.2	NCBI
	TAAR 9-like LOC100148476	10:41973844..41974831	NC_007121.5	NCBI
	Dr_TAAR6-like LOC100332366	13:295967..296969	NC_007124.5	NCBI
	Dr_TAAR9-like LOC100000110	13:300938..301940	NC_007124.5	NCBI
**Tetraodon**	Tn_TAAR1(6 of 15)	Un:85474851..85475831	ENSTNIT00000003225	Ensembl
Tetraodon nigroviridis	Tn_TAAR1(11 of 15)	Un:85467788..85468786	ENSTNIT00000002494	Ensembl
	Tn_TAAR1(14 of 15)	Un:35318087..35319082	ENSTNIT00000001436	Ensembl
	Tn_TAAR1(13 of 15)	Un:70258269..70259261	ENSTNIT00000007318	Ensembl
	Tn_TAAR1(15 of 15)	Un:70265697..70266692	ENSTNIT00000007319	Ensembl
	Tn_TAAR1(10 of 15)	Un: 85499769..85500764	ENSTNIT00000008083	Ensembl
	Tn_TAAR1(12 of 15)	14:1075051..1076047	ENSTNIG00000001506	Ensembl
	Tn_TAAR1(5 of 15)	14:1025315..1026317	ENSTNIG00000019179	Ensembl
	Tn_TAAR1(2 of 15)	14:1062370..1063270	ENSTNIG00000001657	Ensembl
	Tn_TAAR1(3 of 15)	14:1072819..1073800	ENSTNIG00000001360	Ensembl
	Tn_TAAR1(9 of 15)	Un:85495812..85496808	ENSTNIG00000005193	Ensembl
**Stickleback**	Ga_TAAR1(2 of 5)	groupXVIII:864898..865894	ENSGACG00000004284	Ensembl
Gasterosteus aculeatus	Ga_TAAR1(5 of 5)	groupXVIII:849379..850381	ENSGACG00000004279	Ensembl
	Ga_TAAR1(1 of 5)	groupXVIII:856337..857339	ENSGACG00000004280	Ensembl
	Ga_TAAR1(4 of 5)	groupXVIII:806425..807427	ENSGACG00000004274	Ensembl
	Ga_TAAR1(3 of 5)	groupI:27258528..2725921	ENSGACG00000015361	Ensembl
**Anole Lizard**	Ac_TAAR1	Un:4463430..4464437	XM_003223314.1	NCBI
Anolius carolinensis	Ac_TAAR2	Un:4474066..4475097	XM_003223315.1	NCBI
	Ac_TAAR5	Un:4484212..4485264	XM_003223316.1	NCBI
**American Alligator**	Am_TAAR1	Un:220473..221480	XM_006270537.1	NCBI
Alligator mississippiensis	Am_TAAR2	245497..246489	XM_006270538.1	NCBI
	Am_TAAR4-like LOC102559019	286493..287536	XM_006270529.1	NCBI
	Am_TAAR4-like LOC102558785	269855..270901	XM_006270528.1	NCBI
	Am_TAAR5-like LOC102561336	297408..298403	XM_006270539.1	NCBI
	Am_TAAR7a-like LOC102561788	335295..336326	XM_006270540.1	NCBI
	Am_TAAR9-like LOC102562260	358195..359220	XM_006270542.1	NCBI
	Am_TAAR9-like LOC102559257	305709..306737	XM_006270530.1	NCBI
**African Coelacanth**	Lc_TAAR4-like LOC102365654	Un:7900..8919	XM_006014231.1	NCBI
Latimeria chalumnae	Lc_TAAR4-like LOC102362127	Un:6731..7825	XM_006014219.1	NCBI
	Lc_TAAR9-like LOC102346275	Un:226..1260	XM_006014034.1	NCBI
	Lc_TAAR4-like LOC102346012	Un:4621..5655	XM_006014033.1	NCBI

	Lc_TAAR4-like LOC102362844	Un:16272..17312	XM_006014013.1	NCBI
	Lc_TAAR4-like LOC102358308	Un:34650..35687	XM_006013795.1	NCBI
	Lc_TAAR4-like LOC102346983	Un:43349..44395	XM_006013765.1	NCBI
	Lc_TAAR4-like LOC102362653	Un:81244..82287	XM_006013513.1	NCBI
	Lc_TAAR5-like LOC102362390	Un:53536..54543	XM_006013512.1	NCBI
	Lc_TAAR4-like LOC102361509	Un:24914..25954	XM_006013443.1	NCBI
	Lc_TAAR4-like LOC102358939	Un:98526..99563	XM_006013436.1	NCBI
	Lc_TAAR4-like LOC102367115	Un:100058..101794	XM_006013007.1	NCBI
	Lc_TAAR4-like LOC102366851	Un:54232..56373	XM_006013006.1	NCBI
	Lc_TAAR4-like LOC102366590	Un:17939..19252	XM_006013005.1	NCBI
	Lc_TAAR4-like LOC102364694	Un:107680..108717	XM_006012998.1	NCBI
	Lc_TAAR5-like LOC102348717	Un:134193..135206	XM_006011945.1	NCBI
	Lc_TAAR5-like LOC102348210	Un:47880..48881	XM_006011943.1	NCBI
	Lc_TAAR1-like LOC102360536	Un:202003..203031	XM_006010896.1	NCBI
**Elephant Shark**	Cm_contig_19181 - ORF 1 (frame 3)	Un:6852..7886	AAVX02019181.1	NCBI
Callorhinchus milii	Cm_contig_19181 - ORF 4 (frame 1)	Un:46282..47310	AAVX02019181.1	NCBI
**Atlantic Salmon**		21359..22306	AGKD01000046	ASalBase/NCBI
Salmo salar		16565..17527	AGKD01000061	ASalBase/NCBI
		35874..36836	AGKD01000272	ASalBase/NCBI
		6099..7052	AGKD01000449	ASalBase/NCBI
		2040..3002	AGKD01001626	ASalBase/NCBI
		16596..17558	AGKD01003248	ASalBase/NCBI
		13148..14107	AGKD01003637	ASalBase/NCBI
		9633..10595	AGKD01004261	ASalBase/NCBI
		12524..13471	AGKD01004637	ASalBase/NCBI
		7621..8583	AGKD01006407	ASalBase/NCBI
		8332..9279	AGKD01009924	ASalBase/NCBI
		9151..10113	AGKD01016613	ASalBase/NCBI
		3006..3971	AGKD01019372	ASalBase/NCBI
		15867..16829	AGKD01020184	ASalBase/NCBI
		10125..11087	AGKD01034652	ASalBase/NCBI
		859..1890	AGKD01044192	ASalBase/NCBI
		796..1821	AGKD01064135	ASalBase/NCBI
		1778..2743	AGKD01072494	ASalBase/NCBI
		2586..3548	AGKD01073835	ASalBase/NCBI
		3202..4164	AGKD01081184	ASalBase/NCBI
		1874..2911	AGKD01084249b	ASalBase/NCBI
		2433..3392	AGKD01089915	ASalBase/NCBI
		2628..3659	AGKD01103530	ASalBase/NCBI
		5286..6239	AGKD01115415	ASalBase/NCBI
		717..1823	AGKD01134375	ASalBase/NCBI
		9267..10224	AGKD01195896*	ASalBase/NCBI

		731..1693	AGKD01495072	ASalBase/NCBI
**OUTGROUPS**	Dr_Histamine Receptor H2	14:1618017..1619248	NM_001045338.1	NCBI
	Hs_Histamine Receptor H2	5:175084847..175136239	NM_001131055.1	NCBI
	Hs_Rhodopsin	3:129247482..129254187	NM_000539.3	NCBI
	Dr_Adrenergic Receptor Beta 3a	8:38812340..38850902	NM_001128335.1	NCBI
	Hs_Adrenergic Receptor Beta 3	8:37820513..37824184	NM_000025.2	NCBI
	Gg_Histamine Receptor H2	13:9783231..9794177	XM_004944905.1	NCBI
	Dr_Dopamine receptor D2a	15:21008238..21035946	NM_183068.1	NCBI
	Hs_Dopamine receptor D2	11:5001..70685	NM_000795.3	NCBI
	Gg_Dopamine receptor D2	24:5748287..5753356	NM_001113290.1	NCBI
	Dr_Serotonin receptor 1A b	21:19778036..19779277	NM_001145766.1	NCBI
	Hs_Serotonin receptor 1A	5:63255560..63257804	NM_000524.3	NCBI
	Gg_Serotonin receptor 1A	Z:19853067..19854401	NM_001170528.1	NCBI

*Contig with partial sequence. Complete TAAR was found in scaffold jcf2339333987_2792921-4584714_ssa21 (Lien et al., in preparation), which contains this contig
