# Supplementary material for: Genomic Organization and Evolution of the Trace Amine-Associated Receptor (TAAR) Repertoire in Atlantic Salmon (Salmo salar)
Source: G3 (Bethesda). 2014 Apr 22;4(6):1135–41. doi: 10.1534/g3.114.010660 (PMC4065256; doi:10.1534/g3.114.010660)
Supplement: Supporting Information [file supp_g3.114.010660_TableS3.pdf]

Table S3 Summary of putatively functional Atlantic salmon TAAR genes including predicted nucleotide length and physical/genetic location. Superscripts denote the various mapping methods utilized.

Contig	Length (bp)	Physical Location	Genetic Location
AGKD01000046	948	unmapped	unmapped
AGKD01000061	963	unmapped	unmapped
AGKD01000272	963	unmapped	unmapped
AGKD01000449	954	unmapped	unmapped
AGKD01001626	963	fps943[1]	Ssa21[1,2]
AGKD01003248	963	fps508[1]	Ssa21[1]
AGKD01003637	960	fps508[1]	Ssa21[1,2]
AGKD01004261	963	unmapped	unmapped
AGKD01004637	948	unmapped	Ssa13[2,3]
AGKD01006407	963	unmapped	Ssa21[2]
AGKD01009924	948	unmapped	unmapped
AGKD01016613	963	fps508[1]	Ssa21[1,2]
AGKD01019372	966	unmapped	Ssa21[2]
AGKD01020184	963	fps508[1]	Ssa21[1,2]
AGKD01034652	963	unmapped	unmapped
AGKD01044192	1026	fps2319[1]	Ssa06[1,3]
AGKD01064135	1026	unmapped	unmapped
AGKD01072494	966	unmapped	Ssa02[2]
AGKD01073835	963	fps943[1]	Ssa21[1,3]
AGKD01081184	963	fps508[1]	Ssa21[1,2]
AGKD01084249b	1038	fps798[1]	Ssa15[1,2]
AGKD01089915	960	fps943[1]	Ssa21[1,3]
AGKD01103530	1026	unmapped	unmapped
AGKD01115415	954	fps508[1]	Ssa21[1,2]
AGKD01134375	1038	fps798[1]	Ssa15[1,2]
AGKD01195896	957	fps508[1]	Ssa21[1]
AGKD01495072	963	unmapped	Unmapped

[1] BLASTn against BAC ends
[2] BLASTn against SNP mapped
[3] Microsatellite marker
