# Supplementary material for: Genomic Organization and Evolution of the Trace Amine-Associated Receptor (TAAR) Repertoire in Atlantic Salmon (Salmo salar)
Source: G3 (Bethesda). 2014 Apr 22;4(6):1135–41. doi: 10.1534/g3.114.010660 (PMC4065256; doi:10.1534/g3.114.010660)
Supplement: Supporting Information [file supp_g3.114.010660_TableS4.pdf]

Table S4 Summary of putative Atlantic salmon TAAR pseudogenes including physical/genetic location and predicted cause of pseudogenziation. Superscripts denote the various mapping methods utilized.

Name	Physical Location	Genetic Location	Predicted Cause
AGKD01001951	fps508[1]	Ssa21[1,2]	Indel / Frameshift Mutation
AGKD01036711	fps508[1]	Ssa21[1,2]	Indel / Frameshift Mutation
AGKD01087949	fps508[1]	Ssa21[1,2]	Partial match
AGKD01040598	fps798[1]	Ssa15[1,2]	Partial match
AGKD01084249a	fps798[1]	Ssa15[1,2]	Partial match
AGKD01222984	fps798[1]	Ssa15[1,2]	Nonsense mutation
AGKD01000376	unmapped	unmapped	Indel / Frameshift Mutation
AGKD01014617	unmapped	Ssa21[2,3]	Indel / Frameshift Mutation
AGKD01024914	unmapped	unmapped	Partial Match
AGKD01052335	unmapped	unmapped	Nonsense Mutation
AGKD01073505	unmapped	Ssa14[3]	Indel / Frameshift Mutation
AGKD01074920	unmapped	Ssa21[2]	Nonsense Mutation
AGKD01084072	unmapped	Ssa21[2]	Indel / Frameshift Mutation
AGKD01085575	unmapped	unmapped	Indel / Frameshift Mutation
AGKD01102951	unmapped	unmapped	more sequence data required
AGKD01137574	unmapped	Ssa06[2]	Partial Match
AGKD01143659	unmapped	unmapped	more sequence data required
AGKD01145155	unmapped	unmapped	more sequence data required
AGKD01162248	unmapped	Ssa04[2]	Partial Match
AGKD01165582	unmapped	unmapped	Frameshift Mutation / Nonsense Mutation
AGKD01284261	unmapped	Ssa01[2]	Partial Match
AGKD01437520	unmapped	Ssa21[2]	more sequence data required
AGKD01455862	unmapped	Ssa21[2]	Indel / Frameshift Mutation
AGKD01503373	unmapped	unmapped	more sequence data required
AGKD01507155	unmapped	unmapped	Partial Match

[1] BLASTn against BAC ends
[2] BLASTn against SNP mapped
[3] Microsatellite marker
